# A *Calsequestrin-1* Mutation Associated with a Skeletal Muscle Disease Alters Sarcoplasmic Ca^2+^ Release

**DOI:** 10.1371/journal.pone.0155516

**Published:** 2016-05-19

**Authors:** Maria Cristina D’Adamo, Luigi Sforna, Sergio Visentin, Alessandro Grottesi, llenio Servettini, Luca Guglielmi, Lara Macchioni, Simona Saredi, Maurizio Curcio, Chiara De Nuccio, Sonia Hasan, Lanfranco Corazzi, Fabio Franciolini, Marina Mora, Luigi Catacuzzeno, Mauro Pessia

**Affiliations:** 1 Section of Physiology & Biochemistry, Department of Experimental Medicine, University of Perugia School of Medicine, Perugia, Italy; 2 Department of Cell Biology and Neuroscience, Istituto Superiore di Sanità, Roma, Italy; 3 SuperComputing Applications and Innovation–CINECA, Roma, Italy; 4 Department of Chemistry, Biology and Biotechnology, University of Perugia, Perugia, Italy; 5 Neuromuscular Diseases and Neuroimmunology Unit, Foundation IRCCS Neurological Institute C. Besta, Milano, Italy; 6 Department of Physiology & Biochemistry, Faculty of Medicine and Surgery, University of Malta, Msida, Malta; Cinvestav-IPN, MEXICO

## Abstract

An autosomal dominant protein aggregate myopathy, characterized by high plasma creatine kinase and *calsequestrin-1* (CASQ1) accumulation in skeletal muscle, has been recently associated with a missense mutation in *CASQ1* gene. The mutation replaces an evolutionarily-conserved aspartic acid with glycine at position 244 (p.D244G) of CASQ1, the main sarcoplasmic reticulum (SR) Ca^2+^ binding and storage protein localized at the terminal cisternae of skeletal muscle cells. Here, immunocytochemical analysis of myotubes, differentiated from muscle-derived primary myoblasts, shows that sarcoplasmic vacuolar aggregations positive for CASQ1 are significantly larger in *CASQ1*-mutated cells than control cells. A strong co-immuno staining of both RyR1 and CASQ1 was also noted in the vacuoles of myotubes and muscle biopsies derived from patients. Electrophysiological recordings and sarcoplasmic Ca^2+^ measurements provide evidence for less Ca^2+^ release from the SR of mutated myotubes when compared to that of controls. These findings further clarify the pathogenic nature of the p.D244G variant and point out defects in sarcoplasmic Ca^2+^ homeostasis as a mechanism underlying this human disease, which could be distinctly classified as “*CASQ1-couplonopathy”*.

## Introduction

A surplus of endogenous proteins within circumscribed areas of muscle fibers often characterizes protein aggregate myopathies. Moreover, chronically elevated plasma creatine kinase (CK) is a common feature of these myopathies and may precede clinical presentation. Patients from several Italian families with hyperCKaemia and clinical myopathy, whose muscle biopsies showed pathological features and sarcoplasmic inclusions positive for the SR Ca^2+^ binding/storage protein calsequestrin-1 (CASQ1), have been previously described [1,2]. The main clinical features of these patients were myalgia, muscle cramps or mild muscle weakness, and mild to moderate elevation of serum CK. Recently, a heterozygous missense mutation in the *CASQ1* gene, involving an evolutionarily-conserved residue of the CASQ1 protein (p.D244G), has been identified [3,4]. Nevertheless, the pathophysiological mechanisms related to this mutation remain mostly unclear.

In mammals, two CASQ isoforms are expressed in skeletal muscle and the heart [5]. CASQ1 is the only isoform expressed in fast-twitch skeletal muscle, and the major isoform expressed in slow-twitch skeletal muscle. CASQ2 is the sole isoform expressed in cardiac muscle and represents a minor component of slow-twitch skeletal muscle [6]. The low affinity, high capacity Ca^2+^-binding protein CASQ1 is concentrated within discrete areas of the muscle SR, such as in the terminal cisternae, where it buffers large amounts of Ca^2+^. The close proximity of these CASQ1 concentrated areas to ryanodine release channels allows for the easy release of this Ca^2+^ pool to the surrounding cytosol giving rise to the [Ca^2+^]_i_ increases that sustain contraction. To support this, CASQ1 ablation in mice results in reduced levels of SR Ca^2+^ release and in altered contractile properties [7,8].

In the present study, we carried out functional investigations of CASQ1-D244G showing that this missense mutation alters the expression pattern of CASQ1 in patient derived myotubes and decreases the release of Ca^2+^ from the SR. This inherited skeletal muscle disease, involving a component of the couplon [9], could be distinctly called “*CASQ1-couplonopathy*”.

## Methods

### Myotubes cell cultures

Patient specimens were obtained from the biobank directed by Dr.Marina Mora. Written, informed consent was obtained from the subjects or their parents/legal guardians. Research was conducted according to protocols approved by the Institutional Review Board of the Besta Neurological Institute and University of Perugia, and in compliance with the Helsinki Declaration and local legislation. Control muscle cell cultures were obtained from patients suspected of neuromuscular disease, but who had normal muscle on biopsies and no *CASQ1* mutations. Primary myoblasts were derived directly from biopsied material by culturing in Dulbecco's modified Eagle’s medium (DMEM; Lonza Group Ltd, Basel, Switzerland) containing 20% heat-inactivated fetal bovine serum (FBS) (Gibco Life Technologies), 1% penicillin-streptomycin (Lonza), L-glutamine (Lonza), 10 μg/ml insulin (Sigma Aldrich, St. Louis, MO), 2.5 ng/ml basic fibroblast growth factor (bFGF) (Gibco Life Technologies), and 10 ng/ml epidermal growth factor (EGF) (Gibco). The medium was changed twice weekly and the cultures examined by inverted-phase microscopy. At 70% confluence they were dissociated enzymatically with trypsin-EDTA (Sigma) and seeded for immediate propagation, or frozen in medium containing 10% DMSO (Sigma) for later propagation or other use.

To obtain myotubes, the myoblasts were seeded into 35 mm dishes or in chamber slides in DMEM proliferating medium. At 70% confluence, proliferating medium was changed to differentiating medium (DMEM, 1% penicillin-streptomycin, L-glutamine and insulin, without FBS or growth factors) and the myoblasts were allowed to differentiate into myotubes over 7–9 days [10].

### Immunocytochemistry

Myotubes were fixed in methanol, permeabilized in PBS plus 0.15% Triton-X100 (Bio-Rad, Hercules, CA, USA), for 15 min, incubated in 3% BSA (Bovine serum albumin, Sigma Aldrich, Saint Louis, MO, USA) in PBS for 30 min, followed by a 120 min incubation in anti-CASQ1 (polyclonal antibody Abcam, Cambridge, UK) diluted 1:500 in PBS and 60 min incubation in Alexa Fluor 488 goat anti-rabbit IgG (Molecular probes, Carlsbad, CA, USA) diluted 1:1500 in PBS. All incubations were carried out at room temperature. In patient muscle, CASQ1 was co-localized with RYR1 following incubation of sections with polyclonal anti-CASQ1 (Abcam; 1:500) plus monoclonal anti-RYR1 (Thermo Fischer Scientific, Waltham, MA USA; diluted 1:10), followed by incubation with Alexa Fluor 488 goat anti-rabbit IgG (Molecular Probes Inc, Eugene, OR, USA), and successively by incubation with Alexa Fluor 546 goat anti-mouse IgG (Molecular Probes). Cells and muscle sections were examined under a Zeiss fluorescence microscope or a Leica confocal microscope equipped with hybrid and argon lasers.

### Quantitations of CASQ1-positive puncta

CASQ1-positive puncta were quantitated by using ImageJ 1.47 software (http://rsb.info.nih.gov/nih-image/), on pictures of myotubes from 2 patients and 2 controls, taken at 60x magnification. Briefly, by using the software, a threshold was applied to each picture to obtain black and white images, with areas positive for CASQ1 puncta in black and areas negative in white. The total area occupied by the CASQ1-positive puncta was measured and divided by the number of puncta present in the picture to calculate the average area of puncta. A total of 87 pictures from control and 133 from patient cultures were evaluated. Manual corrections were sometimes applied to eliminate non-myotube/non-CASQ1 punctum areas recognized by the software. The ratio of total area of puncta in a myotube to myotube area (determined as mentioned above) was also calculated, in which case 40 control and 40 patient myotubes were evaluated. Blind measurements were performed by two independent observers.

### Western blotting

40 μg of protein extracted from myotube cultures at 10 days were loaded in a gradient precast gel 4–15% (Bio-Rad) and transferred onto nitrocellulose membranes (Schleicher and Schuell Inc., Keene NH, USA). Membranes were probed with one of the following antibodies: mouse monoclonal anti-caveolin 3 (BD Biosciences, Franklin Lakes, New Jersey), diluted 1:2500 and used as an indicator of how much muscle protein was loaded; rabbit polyclonal anti-CASQ1 (Abcam) diluted1:1000; and mouse monoclonal anti-Ryanodine receptor (Thermo Fischer Scientific) diluted 1:800. An appropriate biotin-conjugated secondary antibody was then applied (Jackson ImmunoResearch, West Grove, PA, USA), followed by peroxidase-conjugated streptavidin (Jackson ImmunoResearch), and detection with SuperSignal West Pico Trial kit (Thermo Fischer Scientific). Western blot bands were quantitated densitometrically using ImageJ, and normalized to caveolin 3 bands in duplicates of cultures from two patients and two controls.

### Molecular modeling and visualization

The structure of the CASQ1 molecule was modelled using, as starting point, the crystal structures of human [11] (PDB code: 3UOM) and rabbit [12] (PDB code: 3TRQ) CASQ1. The D244G mutation was generated in silico using VMD software (Visual Molecular Dynamics). Further molecular structure optimization was performed by energy minimization procedure as described previously [13]. For structural comparison with experimental data, a new crystal structure of the human D244G mutant at high Ca^2+^ concentration was used [14] (PDB code: 5CRG). Root mean square deviation (RMSD) of the CASQ1 fold between our model of D244G and the new crystal structure was 1.75 Å (Cα positions). In particular, superimposition of the mutation site gave the lowest RMSD (0.195 Å) when the residue range 241–265 was used.

### Electrophysiological studies on myotubes

Action potentials (APs) were recorded from myotubes by using the whole-cell configuration of perforated patch under current-clamp mode. Typically, a hyperpolarizing current injection (in the -200 to -800 pA range; 500 ms duration) followed by a pulse to zero current elicited rebound APs. In order to assess the AP shape, the following parameters were determined: Vr: resting membrane potentials; AP_ampl_: action potential amplitude (AP voltage peak minus Vr); AP_dur_: action potential duration at half amplitude; AHP_ampl_: after-hyperpolarization amplitude; AHP_dur_: after-hyperpolarization duration at half amplitude. Depolarization-evoked tail currents (SK currents) due to small conductance Ca^2+^-activated K^+^ channels were obtained from perforated patch, whole-cell voltage-clamp recordings of myotubes derived from 3 patients and 3 controls after culturing myoblasts in differentiation medium for 7–9 days. Recordings were performed as previously described [15]. Currents and voltages were recorded with a HEKA EPC-10 amplifier (List Medical, Darmstadt, Germany) and analyzed with the Patch Master package (version 2X60, Elektronik) and Origin 8.0 software (Microcal Software, USA). Macroscopic currents were filtered at 3 kHz and sampled at 100 μs intervals. SK current densities (ratio of current to capacitance pA/pF) were calculated 0.5 s after the onset of the repolarization pulse back to -40 mV, which was preceeded by depolarizing pulses that lasted 1 s. Depolarizing pulses were increased in 20 mV steps, starting at -40mV and ending at +120 mV. The selective SK channel blocker apamin was used to assess whether the tail current was mainly due to SK channels. The external solution was (mM): NaCl 106.5, KCl 5, CaCl_2_ 2, MgCl_2_ 2, MOPS 5, glucose 20, Na gluconate 30, pH 7.25. The internal solution was (mM): K_2_SO_4_ 57.5, KCl 55, MgCl_2_ 5, MOPS 10, pH 7.20. Electrical access to the sarcoplasm was achieved by adding amphotericin B (200 μM) to the pipette solution. Access resistances in the range 15–25 MΩ were achieved within 10 min from seal formation, and were actively compensated to about 50%. All chemicals were analytical grade. Amphotericin B (50 mM) (Alomone Labs Jerusalem, Israel) was dissolved in DMSO to make stock solutions which were prepared daily. Maximum DMSO concentration in the working solutions used was about 0.1%. Separate application of 0.1% DMSO had no significant effect on membrane currents. Experiments were carried out at room temperature (18–22°C).

### Measurement of sarcoplasmic Ca^2+^ signals

#### Depolarization-induced Ca^2+^ signals

Measurements of the sarcoplasmic Ca^2+^ signals evoked by a depolarizing stimulus were performed using the video imaging recording technique with Fluo-4 AM Ca^2+^ indicator (Invitrogen) in combination with patch-clamp. Fluo-4 AM was dissolved in DMSO at a concentration of 1 mM. Myotubes, differentiated from myoblasts on poly-lysine-coated Petri dishes, were loaded with Fluo-4 AM (5 μM) by incubation in the dark at 37°C (30 min) using an incubation solution having the following composition (in mM): 140 NaCl, 5 KCl, 2 CaCl_2_, 2 MgSO_4_, 10 glucose, and 5 MOPS, pH 7.4, supplemented with 0.05% bovine serum albumin. Myotubes were then placed under an inverted microscope (Axio observed D1, Zeiss) and the patch-perforated configuration was achieved using the methods and solutions described above. CdCl_2_ 100 μM was added to the extracellular solution 10 min before starting the experiments. After selecting the myotube in phase contrast visual field and establishing the perforated patch configuration, an excitation wavelength of 494 nm was continuously applied by means of a mercury short-arc lamp and the emitted light (510 nm) was recorded by a CCD digital camera (AxioCam ICm1) connected to a computer. Ca^2+^ transients obtained by stimulating the myotube with a 2 s long depolarizing stimulus from -60 to +80 mV were recorded. The MatLab software (MathWorks) was used for image analysis, consisting of mean pixel intensity assessment of a selected region of the patch-clamped myotube. Origin 8.0 software (Microcal Software, USA) was used for further analysis and graphical presentation. Fluorescence changes were expressed as the ratio (F/F_0_) of the emitted fluorescence intensity (F) to that recorded just before the depolarizing stimulus (F_0_).

#### Caffeine-induced Ca^2+^ signals

Sarcoplasmic Ca^2+^ was also measured using the Fura-2 AM Ca^2+^ indicator (Invitrogen) in combination with video imaging. Fura-2 AM (2.5 mM) was sonicated for 5 min in Pluronic ac/DMSO (1/4) to make a stock solution. Myotubes, differentiated from myoblasts on poly-lysine-coated glass coverslips, were loaded with Fura-2 AM by incubation in the dark (50 min) with 2.5 μM Fura-2 AM dissolved in recording buffer (see below) at room temperature. The myotubes on coverslips were placed in the perfusion chamber on the stage of an inverted microscope (Axiovert 35, Zeiss, Germany) and perfused with buffer (mM: 140 NaCl, 5 KCl, 1 MgCl_2_, 5.5 D-glucose, 10 HEPES/NaOH, room temperature, pH 7.4). Solutions containing various concentrations of caffeine were made using the buffer and replacing NaCl with caffeine in the ratio of 2:1 to maintain osmolarity. A rapid solution exchanger (Bio-Logic, France) allowed the solution bathing the cells to be switched rapidly between control and test solutions. After selecting myotubes in bright visual field they were illuminated at 340 and 380 nm (Polychrome II, Photonics, Germany) and the emitted light (510 nm) was recorded by a CCD digital camera (PCO, Sensicam, Germany) connected to a computer.

The Imaging Workbench software (INDEC Systems, CA, USA) was used for recording and initial data analysis. Origin 8.0 software (Microcal Software, USA) was used for further analysis and graphical presentation. Ca^2+^ concentrations were expressed as the ratio of emissions at 340 nm to 380 nm. Ca^2+^ signal amplitudes were calculated as maximum amplitude during caffeine challenge minus baseline amplitude recorded prior to challenge.

### Statistics

Data are presented as means ± SEM. The significance of differences between groups was investigated by Student’s *t*-test, and the following levels of significance noted: * *p<*0.05; ** p<0.01; *** p<0.001.

## Results

### CASQ1-positive aggregates in myotubes carrying the D244G mutation

**Fig 1:**
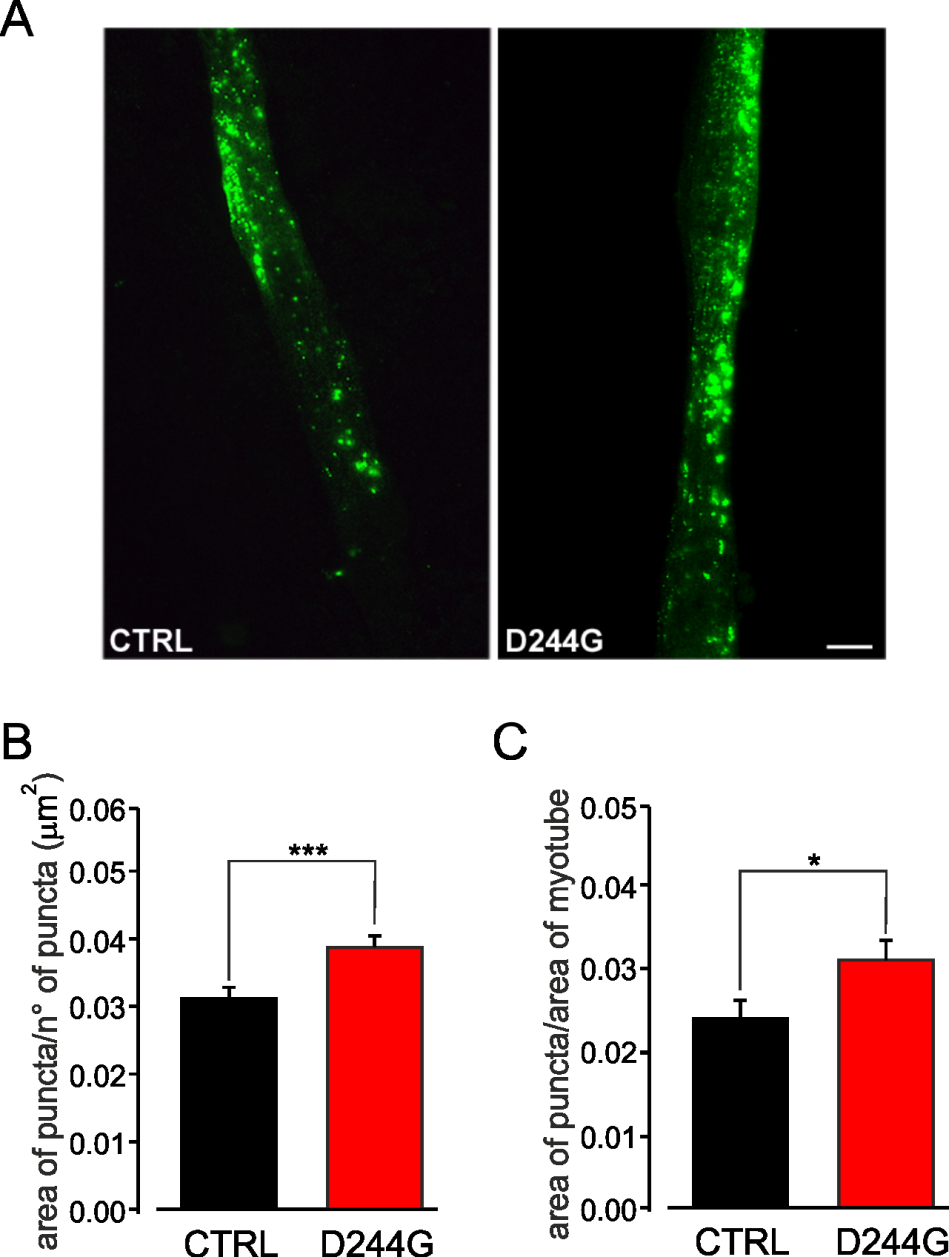
Sarcoplasmic inclusions of CASQ1 in myotubes. (**A**) Immunocytochemical staining of CASQ1-positive puncta in control (*left panel*) and patient (*right panel*) myotubes (scale bar = 10 μm). (**B**) Averaged area of CASQ1-positive puncta in control and patient myotubes expressed as: total area of puncta(μm^2^)/n°of puncta. (**C**) Average ratio of CASQ1-positive puncta in control and patient myotubes expressed as total area of puncta (μm^2^)/total area of myotube (μm^2^). The data are mean±SEM (*p<0.05, ***p<0.001).

To establish a cellular model suitable to investigate the mechanisms underlying this CASQ1-couplonopathy in a physiological setting, primary myoblasts were derived directly from biopsied healthy subjects (WT) and patients carrying the D244G mutation and allowed to differentiate into myotubes (Fig 1A). Immunocytochemical analysis of cell samples showed that the average area of CASQ1-positive puncta was significantly larger in patient than control myotubes (0.039±0.015 μm^2^ *vs* 0.031±0.008 μm^2^; p<0.001; Fig 1B). The average ratio of total area of puncta in a myotube to myotube area was also significantly greater in patient than control cells (0.031±0.02 *vs* 0.02±0.01; p<0.05; Fig 1C).

**Fig 2:**
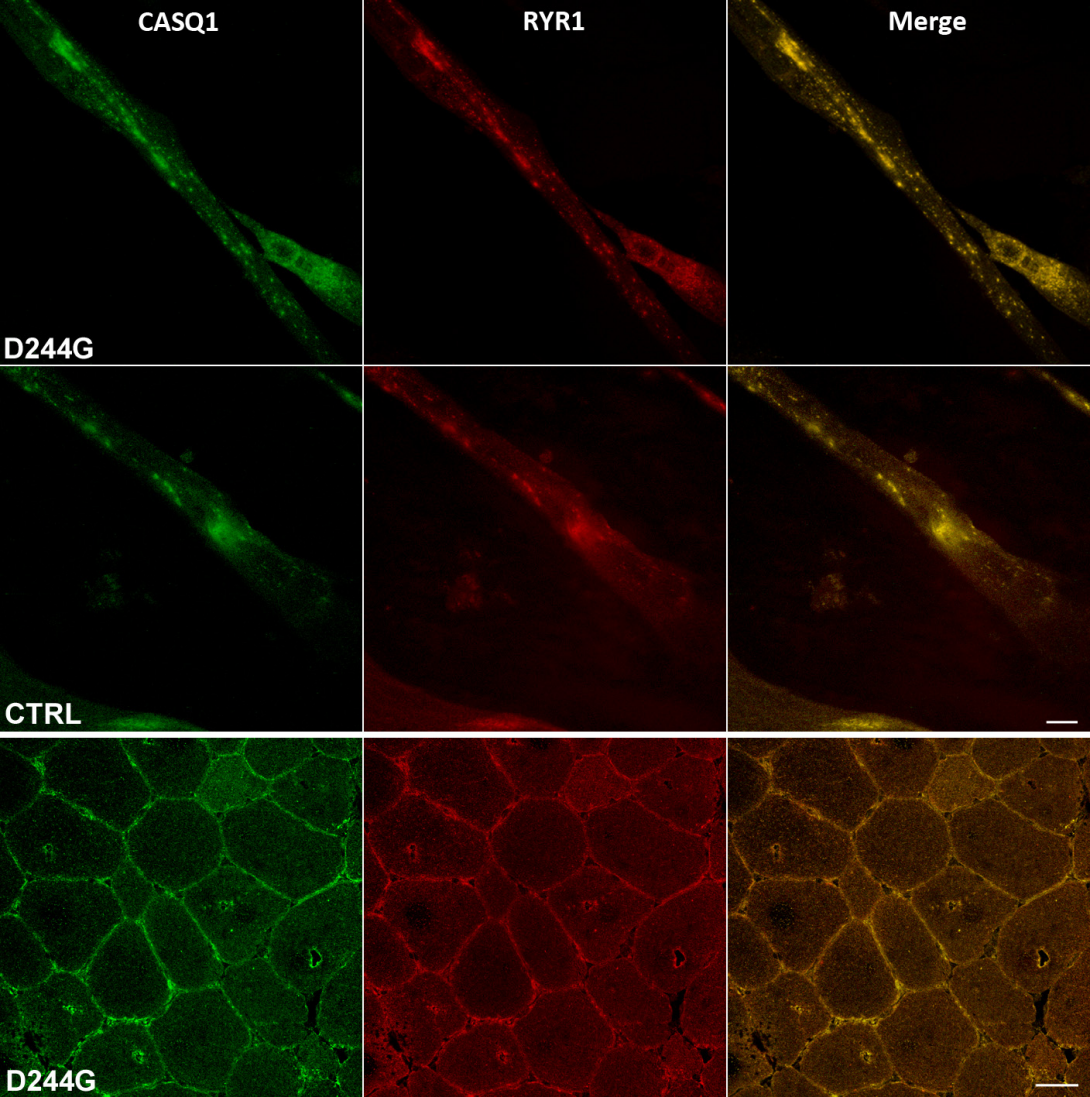
Confocal images of CASQ1 and RYR1 co-localization in both myotube and muscle biopsy. Immunocytochemical staining of RYR1 and CASQ1 in patient (*upper panels*) and control myotubes (intermediate panels; scale bar = 10 μm). The lower panels show CASQ1 and RYR1 co-staining in vacuoles of fibers derived from a patient muscle biopsy (scale bar = 50 μm).

Immunocytochemical staining of RyR1 and CASQ1 in patient and control myotubes showed that both proteins co-localized within puncta (Fig 2). A strong co-immuno staining of both RYR1 and CASQ1 was also noted in the vacuoles of muscle biopsies derived from patients (Fig 2).

Densitometric analysis of the immunoblot bands of protein extracted from myotube cultures showed that CASQ1 values were higher in patient samples compared to controls (1.12±0.21 *vs* 0.62±0.48, respectively), whereas, values for RyR1 bands were similar (0.26±0.22 *vs* 0.21±0.11, respectively). Collectively, these data indicate that patient-derived cultured myotubes recapitulate distinct morphological features of diseased adult muscle fibers, namely, a broader presence of CASQ1 aggregates, as we have previously shown by confocal microscopy analysis of samples biopsied from the same patients [3], and the co-expression of RYR1 and CASQ1 in muscle vacuoles (Fig 2).

### Structural analysis of WT and mutated CASQ1

**Fig 3:**
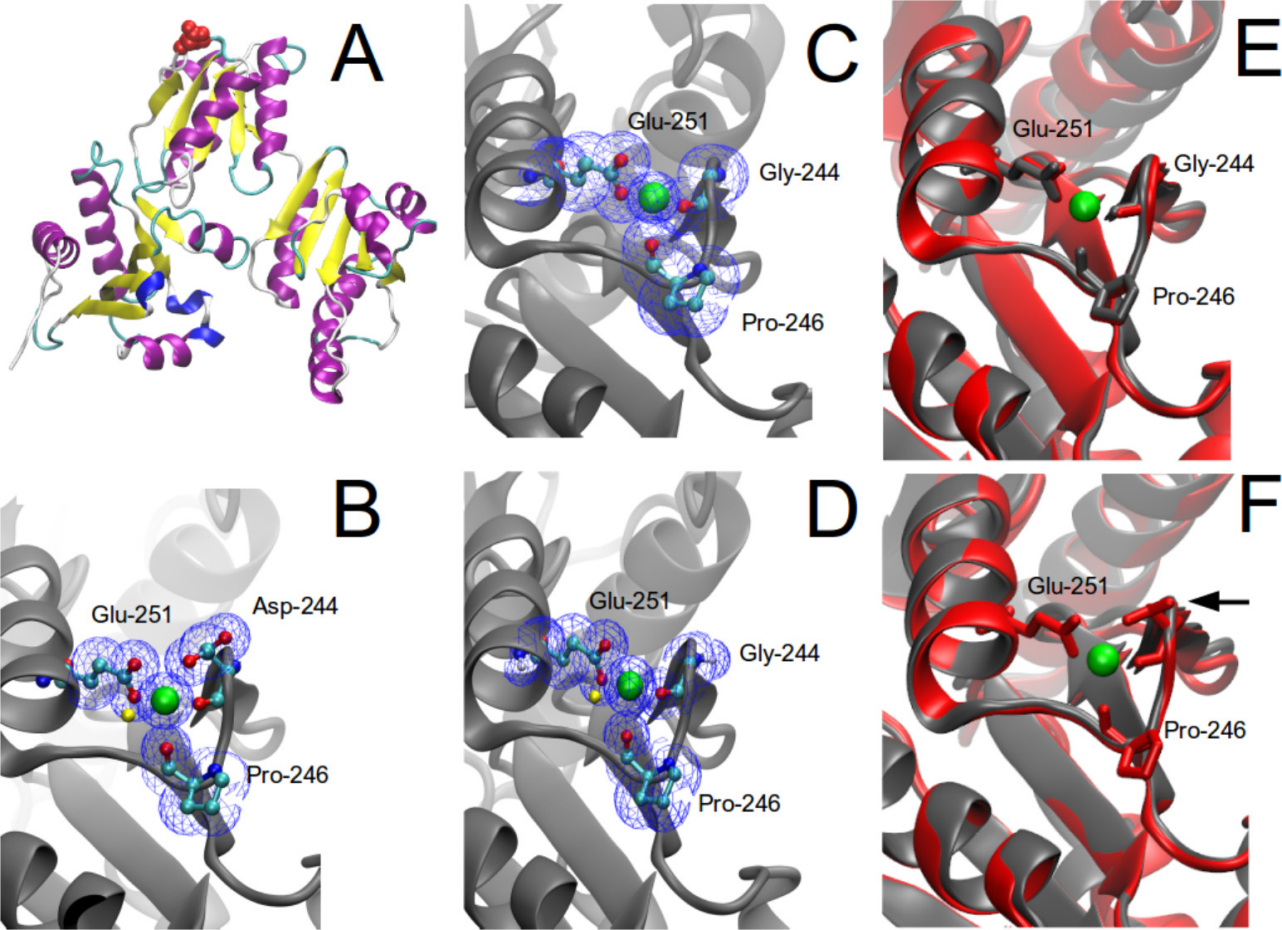
Molecular modeling of CASQ1. (**A**) Ribbon representation of a CASQ1 monomer (*the red spheres indicate the D244 residue*). (**B**) Close up view of the region of interest in WT protein. A Ca^2+^ ion (*green sphere*) is coordinated by the carbonyl oxygen of the backbone and the carboxylic acid group of the side chain of aspartate 244 (*oxygen atoms and water molecule are shown as red and* yellow *spheres*, *respectively*). Two other residues (glutamate E251 and proline 246) also coordinate the same Ca^2+^. (**C**) Homology modelling of CASQ1 in which the aspartate 244 was replaced by a glycine (*see Methods*). (**D**) Crystal structure of CASQ1 carrying the D244G mutation determined by X-Ray diffraction (*see text*) [13]. (**E**) Superimposition of the homology model (*grey*) and the crystal structure (*red*) for CASQ1-D244G protein. (**F**) Superimposition of the homology model (*grey*) and the crystal struxcture (*red*) for CASQ1-WT protein (*the arrow indicates the residue D244*).

Based on the available data from the crystal structure of human CASQ1 [11], we located the aspartate residue at position 244 and used *in silico* mutagenesis to replace it with glycine (Fig 3). The model indicated that a Ca^2+^ ion is coordinated by both the carbonyl oxygen of the peptide backbone and the glutamate carboxylic acid (Fig 3B). This was corroborated by analyzing the X-ray structure of rabbit CASQ1 [12] (*not shown*). Nearby glutamate (E251) and proline (P246) also contributed to Ca^2+^ coordination (Fig 3B). Substitution of glutamate by glycine (D244G) did not obviously alter the ability of the backbone carbonyl oxygen to coordinate Ca^2+^, yet it eliminated the carboxylic side-chain and its contribution to Ca^2+^ coordination (Fig 3C). A recent study reported the crystal structure of the D244G mutant [14]. Using this new experimental data (Fig 3D), we validated our model superimposing the two structures (*model and crystal structure;* Fig 3E) and corroborated our initial prediction on the structural effect of the D244G mutation. Indeed, the RMSD of the Cα carbon atoms of the superimposed homology model and crystal structures for D244G resulted in a 1.75 Å difference in the overall structure, whilst at the mutation site the structural difference was 0.195 Å (residue ranging from 241–265). We have also compared the RMSD of the Cα carbon atoms of the D244G model with that of WT X-Ray structure and found that the model differed 1.20 Å in the overall structure and 0.163 Å in the region where the mutation is located (residue ranging from 241–265) (Fig 3F). Ovearll, this analysis indicated that the conformation of the homology model and crystal structure in the region of interest is highly similar. Furthermore, these results and published data suggest that the D244G mutation may alter sarcoplasmic Ca^2+^ homeostasis, although this remains an open question.

### Assessments of electrophysiological properties of myotubes

**Fig 4:**
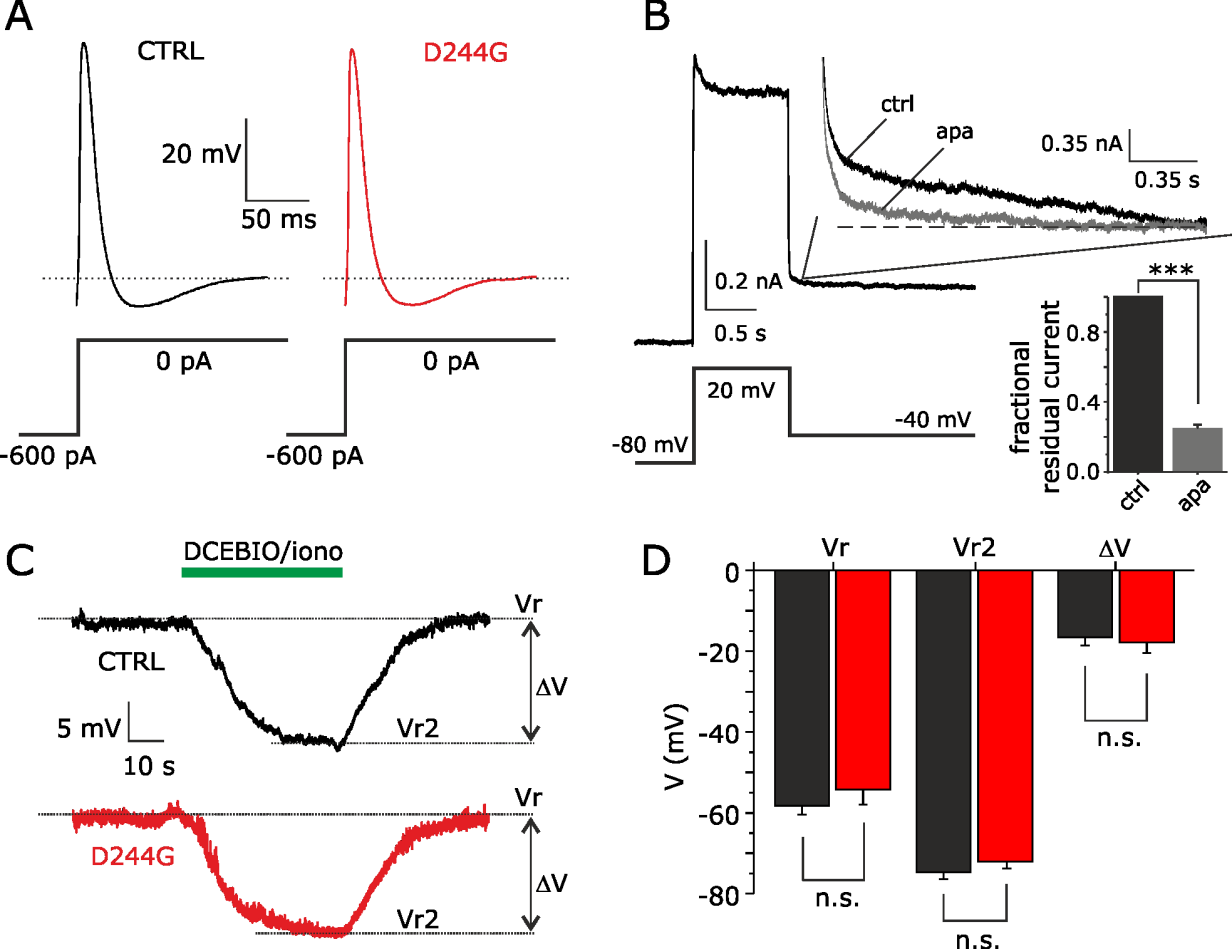
Recordings of action potentials and SK type Ca^2+^-activated K^+^ currents from myotubes. (**A**) Rebound APs elicited by a hyperpolarizing current (-600 pA, 500 ms), followed by a pulse to zero current injection, in sample myotubes from a patient (*red*) and a control (*black*). (**B**) Currents, elicited in a control myotube by a pre-pulse to +20 mV, followed by repolarization to -40 mV. Tail currents are shown enlarged before (*ctrl*) and after apamin (*apa*) application (100 nM). The bar graph (*inset*) shows the fractional tail current before and after apamin application. (**C**) Membrane voltage traces recorded from sample control (*black*) and patient (*red*) myotubes, showing the hyperpolarizing effect of co-application of 100 μM of the SK channel activator DCEBIO and 2 μM of the Ca^2+^ ionophore agent ionomycin (indicated as DCEBIO/iono) in the recording chamber, without applying holding current. (**D**) Plot showing the mean membrane potential before (Vr) and after (Vr2) bath application of DCEBIO/iono, and the mean DCEBIO/iono induced hyperpolarization (ΔV) in control (*black*) and patient (*red*) myotubes. The data are mean±SEM; n = 6.

To investigate D244G-induced dysregulation of sarcoplasmic Ca^2+^ homeostasis we first validated patient derived myotubes as an appropriate model to study the pathogenic mechanisms underlying *CASQ1-couplonopathy*. Perforated patch-clamp recordings from patient and control myotubes were performed to establish their basic electrophysiological properties and assess for cell ability to generate rebound *all-or-none* action potentials (APs), followed by after-hyperpolarization, an index of proper cell differentiation [16]. Fig 4A shows representative APs recorded in current-clamp mode from a WT and D244G myotube, when stimulated by a hyperpolarization-induced current prepulse followed by a step to zero current injection. Analysis of several electrophysiological parameters revealed no statistically significant differences between the resting membrane potential (Vr) and the APs recorded from WT and D244G cells (Table 1).

**Table 1:** Action potential parameters assessed from control and D244G myotubes.

PARAMETERS	CTRL	D244G	P (CTRL vs D244G)
**V**_**r**_ **(mV)**	-48.5±7.1 (5)	-53.1±3.2 (5)	0.5777
**AP**_**ampl**_ **(mV)**	62.5±6.2 (5)	63.8±3.0 (5)	0.8516
**AP**_**rt**_ **(ms)**	11.58±0.5 (5)	11.5±1.5 (5)	0.9485
**AHP**_**ampl**_ **(mV)**	5.8±1.0 (5)	5.5±2 (5)	0.8921
**AHP**_**dur**_ **(ms)**	74.6±11 (4)	61.8±12 (4)	0.4707

Abbreviations: Vr: resting membrane potentials; AP_ampl_: action potential amplitude; AP_dur_: action potential duration; AHP_ampl_: after-hyperpolarization amplitude; AHP_dur_: after-hyperpolarization duration at half amplitude. The data are means±SEMs; the number of observations is indicated in parenthesis.

In voltage-clamp mode, 1 sec depolarization pulses at +20 mV activated outward currents, which were followed by slowly decaying tail-currents upon returning to -40 mV (Fig 4B). The decaying currents were markedly inhibited by apamin, a selective blocker of Ca^2+^-activated K^+^ channels of the SK type (Fig 4B, *enlarged traces*). We next examined the level of surface expression for SK channels in normal and mutated myotubes by using ionomycin, a potent Ca^2+^ ionophore and, DCEBIO an enhancer of SK channels. To attain both maximal intracellular Ca^2+^ concentration and full activation of SK channels, 2 μM ionomycin (in the presence of extracellular Ca^2+^), and 100 μM DCEBIO were co-applied onto myotubes. This procedure resulted in membrane hyperpolarizations of similar degrees in both WT and D244G myotubes (Fig 4C and 4D). These tests showed that some basic electrical properties of myotubes differentiated from either healthy subjects or patients were similar further supporting the myotube as an appropriate model to study the pathogenic mechanisms underlying this myopathy.

### Effects of D244G mutation on SR Ca^2+^ release upon electrical stimulation

**Fig 5:**
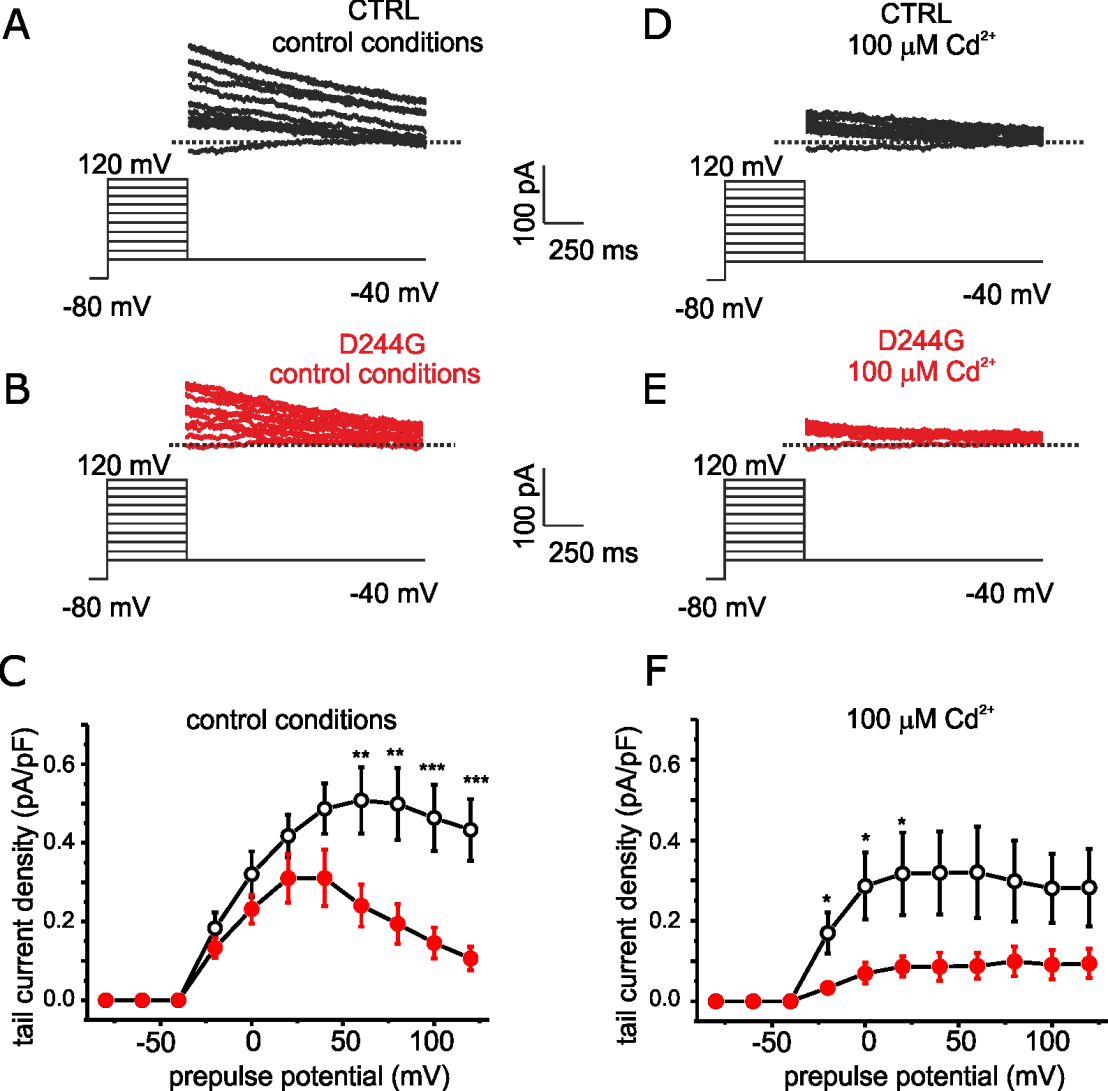
SK tail current densities in control vs D244G expressing myotubes. Sample tail currents elicited by pre-pulse potentials from -40mV to +120mV and recorded at a holding potential of -40 mV from control (**A**, *black traces*) and D244G expressing (**B**, *red traces*) myotubes. (**C**) Tail current densities, calculated from control (*open circles*) and D244G (*red circles*) myotubes and, plotted as a function of pre-pulse potentials. Sample tail currents recorded as described above in the presence of Cd^2+^ (100 μM), from control (**D**, *black traces*) and D244G (**E**, *red traces*) myotubes. (**F**) Tail current densities in the presence of Cd^2+^ (100 μM) that were recorded, calculated and plotted as described in C. Data reported in plots **C** and **F** were obtained from 3 independent experiments (n = 6; *p<0.05; **p<0.01; ***p<0.001).

We took advantage of surface expression of SK channels in myotubes and exploited them as sensors of intracellular Ca^2+^ to explore whether the D244G mutation in CASQ1 alters SR Ca^2+^ release under near physiological conditions (*i*.*e*., electrical stimulation). Patch-clamp recordings of slowly decaying SK tail currents were performed to address this issue. Analysis of SK tail currents showed that they were significantly smaller in patient than control myotubes at most depolarized potentials (Fig 5A–5C). SK currents, recorded under these experimental conditions, may be however activated by both Ca^2+^ influx through plasma membrane voltage-gated Ca^2+^ channels and Ca^2+^ release through RyR channels involved in excitation-contraction coupling. To isolate SK currents, activated by SR’s release of Ca^2+^ through RyR channels, Cd^2+^ was applied to inhibit Ca^2+^ influx through plasma membrane Ca^2+^ channels. Under these conditions SK tail current densities measured from patient myotubes were still significantly smaller than those measured from controls (Fig 5D–5F). Altogether these data suggested that the p.D244G mutation reduces SR Ca^2+^ release during excitation-contraction coupling.

### The D244G mutation reduces Ca^2+^ signals from myotubes

**Fig 6:**
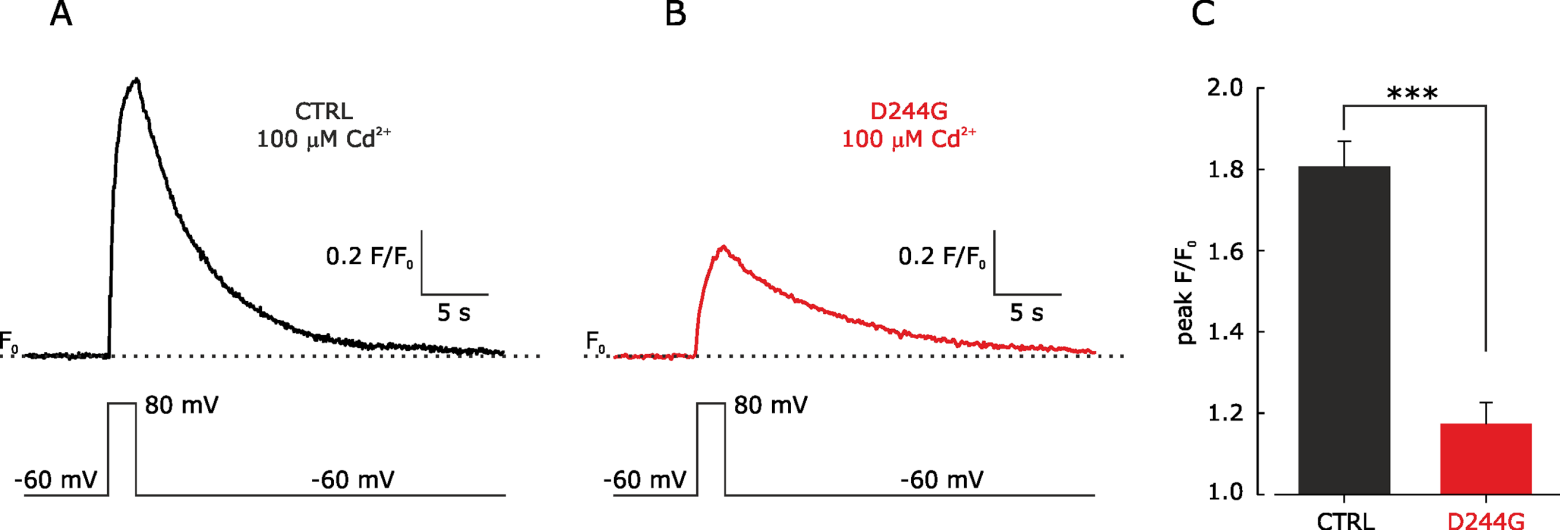
Sarcoplasmic Ca^2+^ signals elicited by depolarization in control vs D244G myotubes. Representative fluorescence traces elicited by depolarization to +80 mV (in the presence of CdCl_2_ 100 μM) from Fluo-4AM-loaded control (**A**, CTRL) and patient (**B**, D244G) myotubes. The voltage-clamp steps applied to myotues are shown below each sample trace. (**C**) Bar graph showing the mean Ca^2+^ signals elicited from control (*black*) and D244G (*red*) myotubes (the data are mean±SEM; n = 5; ***p<0.001).

To provide more direct evidence concerning the effect of the mutation on the amount of Ca^2+^ released from the SR through RyR channels, during the excitation-contraction coupling, we measured Ca^2+^ signals resulting from a brief voltage-clamp step delivered through the patch pipette that depolarized the myotube to +80 mV (Fig 6). In the presence of CdCl_2_ (100 μm), to inhibit Ca^2+^ influx through plasma membrane Ca^2+^ channels, depolarization of a control myotube elicited a Ca^2+^ signal that reached a peak and decayed to basal level after several seconds (Fig 6A). Remarkably, depolarization of myotubes carrying the D244G mutation resulted in much smaller peak Ca^2+^ signals compared to control (Fig 6B and 6C).

**Fig 7:**
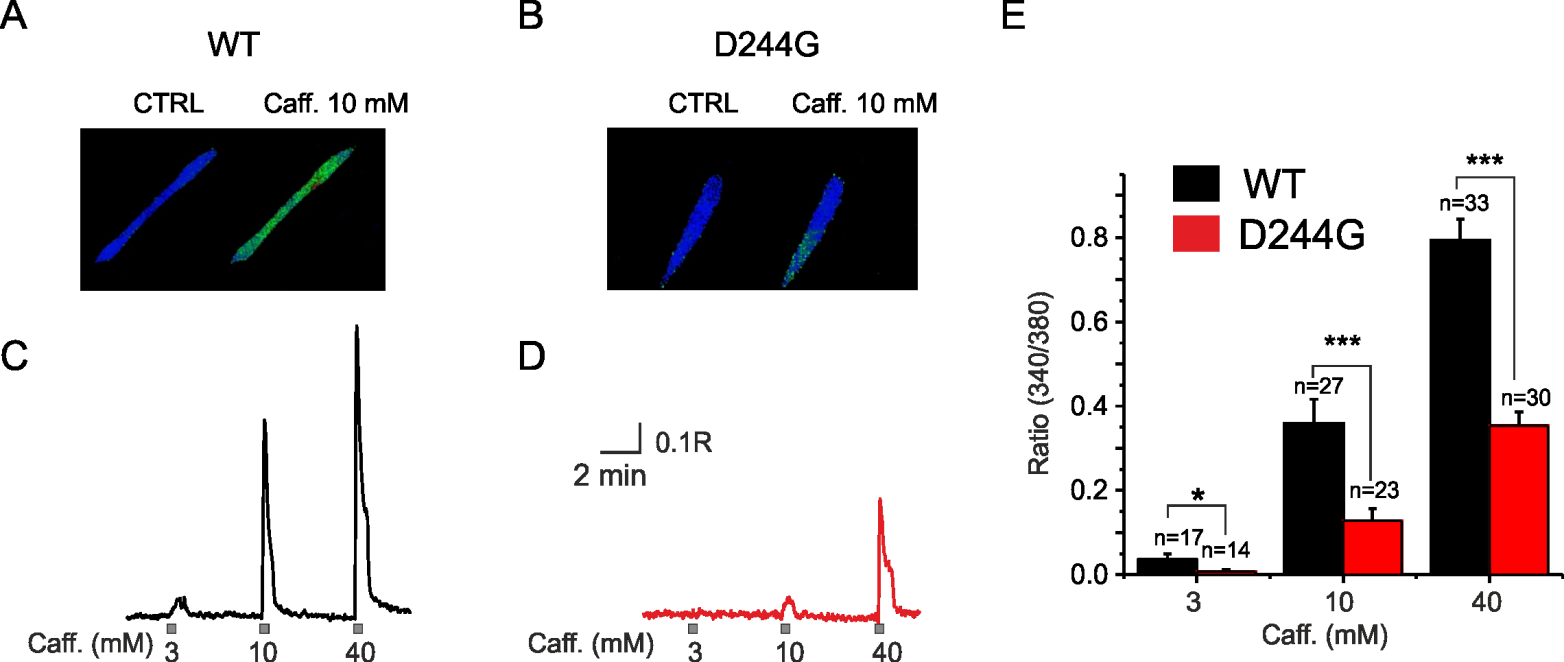
Sarcoplasmic Ca^2+^ signals elicited by caffeine in control vs D244G myotubes. Representative fluorescence images from fura-2-loaded control (**A**) and patient (**B**) myotubes before (CTRL) and during caffeine application (Caff 10 mM). Sample traces showing signals elicited by caffeine (Caff 3, 10, 40 mM) and recorded from myotubes derived from controls (**C**) and patients (**D**). (**E**) Bar graph showing the mean Ca^2+^ signals elicited by different caffeine concentrations from control (*black*) and D244G (*red*) myotubes. Data are means with error bars representing SEMs; the number of myotubes from which Ca^2+^ signals were measured is shown above each bar (*p<0.05; ***p<0.001).

Ca^2+^ signals resulting from application of the RyR agonist caffeine on myotubes were also measured. As shown in Fig 7, exposure of patient myotubes to increasingly elevated levels of extracellular caffeine resulted in Ca^2+^ transients that were of significantly lower amplitude than those induced in control myotubes.

## Discussion

Patients carrying the *D244G* mutation present with a myopathy characterized by one or more symptoms that include episodes of muscle cramping, mild myalgia, muscle weakness, fatigue, and hyperCKaemia. Here, we have presented results that prove that Ca^2+^ dynamics, vital for normal muscular function, are altered and underlie this *D244G* myopathic phenotype.

The substitution of the highly conserved aspartate amino acid for glycine, at position 244 of the CASQ1 polypeptide, results in CASQ1 protein aggregation that manifests as vacuolar inclusions that stain positive for CASQ1 [3]. We have previously shown by means of immunohistochemistry, electron microscopy and electrophoretic investigations that CASQ1 was present in greater quantities in the muscle fibers of patients carrying the mutation when compared to that of control subjects, and that the mutated CASQ1 was responsible for the abnormal CASQ1 inclusions [3]. Here we report that myotubes derived from patients also recapitulate this distinct feature of the disease, namely, the formation of CASQ1 vacuolar aggreagates. In addition we also point out that both CASQ1 and RyR1 coexist in muscle vacuoles, an observation that might be pathophysiologically relevant. Our previous electrophoretic studies showed that the mutated protein does not migrate in the same way as the normal protein and displays the tendency to form complexes [3]. This finding is consistent with the tendency of D244G protein to aggregate abruptly and abnormally, *in vitro* [14].

The carbonyl oxygen atom of Asp244 (the residue mutated in our patients), along with that of Pro-246 and the carboxylate of Glu-251, form a high-affinity Ca^2+^ coordination site in the CASQ1 protein (Fig 3) [11]. Our structural model of native and mutated CASQ1, showing that the substitution of Asp-244 with the more flexible Gly destabilizes a Ca^2+^ coordination site, is fully consistent with the recently reported CASQ1-D244G crystal structure [14]. Lewis *and colleagues* also showed that crystallized D244G under low Ca^2+^ concentrations resulted in a disordered lattice lacking the linear back-to-back interaction characteristic of CASQ1. Under high Ca^2+^ concentrations sufficient for Ca^2+^ binding into the coordination site, the lattice regained order, however without the normal back-to-back contact. The D244G-induced abnormal CASQ1 polymerization and aggregation, and the presence of larger amounts of mutated CASQ1 protein stored in intracellular organelles of diseased muscles (Figs 1 and 2) [3], could lead to sarcomere destabilization and cell toxicity that may eventually contribute to some of the associated muscular symptoms of the disease, such as elevated plasma CK. The overall condition is however clinically mild possibly because compensatory mechanisms/proteins intervene to ensure near normal muscle functionality.

The D244G-induced disruption in CASQ1 polymerization may also affect Ca^2+^ diffusion towards the open Ca^2+^ release channels by altering the local concentration of diffusible Ca^2+^ ions, their one-dimensional directionality or CASQ1-RyR allosteric interactions, overall reducing RyR-mediated Ca^2+^ release from the SR as documented in this study. It has been shown that CASQ1 inhibits RyR channels, depending on its phosphorylation status and whether it is bound to RyR directly or indirectly via anchoring proteins [9,17], all of which could be altered as a consequence of the associated structural change in CASQ1.

That in this study, patient-derived myotubes had large CASQ1 aggregates, greater in area than that in myotubes derived from control subjects and similar to that observed from patient muscle biopsies, make myotubes differentiated from biopsied primary myoblasts an appropriate model to investigate the pathogenic mechanisms underlying this CASQ1-couplonopathy. Having basic electrophysiological properties comparable to healthy myotubes is consistent with the clinically mild phenotype of the disease and further strengthens the usefulness of this model. Activated by and dependent on intracellular Ca^2+^, the SK current makes a useful, albeit indirect, functional assessment tool for investigating mechanisms that underlie SR calcium dysfunction. The reduced SK tail-current densities measured from patient myotubes is functional proof that the mutation results in a physiologically significant reduction in Ca^2+^ release from the SR. A more direct evidence lies in the results from the Ca^2+^ signal recordings where myotube depolarization evoked Ca^2+^ signals of lower amplitude than those evoked in control myotubes. Also the exposure to increasingly elevated levels of caffeine, an RyR agonist, resulted in significantly lower Ca^2+^ transients in patient myotubes. Consistently, the SK tail-current activation results, both from depolarization- and caffeine-induced Ca^2+^ signals, indicate that the CASQ1’s p.D244G mutation disrupts normal muscle function by compromising SR Ca^2+^ release via RyR channels. Noteworthy, caffeine has been widely used to deplete the SR of Ca^2+^ [18], and the amplitude of caffeine-induced Ca^2+^ transients is commonly taken as an index of the SR Ca^2+^ load [19]. Our observations suggesting that D244G could also affect the loading capacity of the SR (*i*.*e*., the amount of Ca^2+^ sequestered by mutated CASQ1), is consistent with data showing that the D244G protein possesses a lower binding capacity *in vitro*, and that the aggregates are less able to bind Ca^2+^ [14].

Overall, the evidence provided here further implicates D244G substitution as pathogenic and points out defects in Ca^2+^ homeostasis within muscle fibers as a possible mechanism underlying muscle cramping, weakness and fatigue. With a much clearer understanding of the D244G mutation and its effect on altering sarcoplasmic Ca^2+^ release, the major challenges to be tackled in the near future would be a better understanding of the mechanisms leading to D244G-induced cell toxicity and designing possible therapeutic interventions for this newly identified CASQ1-couplonopathy.
